# Current approaches and potential challenges in the delivery of gene editing cargos into hematopoietic stem and progenitor cells

**DOI:** 10.3389/fgeed.2023.1148693

**Published:** 2023-09-15

**Authors:** Ramya Murugesan, Karthik V. Karuppusamy, Srujan Marepally, Saravanabhavan Thangavel

**Affiliations:** ^1^ Centre for Stem Cell Research (CSCR), A Unit of InStem Bengaluru, Christian Medical College Campus, Vellore, Tamil Nadu, India; ^2^ Manipal Academy of Higher Education, Manipal, Karnataka, India

**Keywords:** hematopoietic stem cells, gene therapy, gene editing, gene delivery, *in vivo* delivery

## Abstract

Advancements in gene delivery and editing have expanded the applications of autologous hematopoietic stem and progenitor cells (HSPCs) for the treatment of monogenic and acquired diseases. The gene editing toolbox is growing, and the ability to achieve gene editing with mRNA or protein delivered intracellularly by vehicles, such as electroporation and nanoparticles, has highlighted the potential of gene editing in HSPCs. Ongoing phase I/II clinical trials with gene-edited HSPCs for β-hemoglobinopathies provide hope for treating monogenic diseases. The development of safe and efficient gene editing reagents and their delivery into hard-to-transfect HSPCs have been critical drivers in the rapid translation of HSPC gene editing into clinical studies. This review article summarizes the available payloads and delivery vehicles for gene editing HSPCs and their potential impact on therapeutic applications.

## Introduction

Allogenic hematopoietic stem cell transplantation (HSCT) is a standard therapeutic procedure for treating malignancies and inborn errors of the hematopoietic system. From the first bone marrow transplantation (Thomas et al., 1957) to human leukocyte antigen (HLA) identification (Dausset, 1958), HLA-matched bone marrow transplantation (Thomas et al., 1959), and transplantation for the genetic disease X-linked severe combined immunodeficiency (SCID-X1) (Gatti et al., 1968), the therapy has evolved, and over 1.5 million HSCT procedures have been performed worldwide (Niederwieser et al., 2022). Despite the high success rate of allogenic HSCT, the shortage of HLA-matched donors led to the development of autologous HSPC gene therapy (Gorin et al., 1977). The emergence of molecular techniques in the early 1960s, such as the subcloning of mammalian genes into prokaryotic plasmids and bacteriophages, was anticipated as the precursor to human gene therapy. Subsequent studies that revealed viral DNA integration in SV40-transformed cells provided a path for viruses to be used as a delivery vehicle (Sambrook et al., 1968). An empirical investigation of avian and murine oncoretrovirus biology resulted in the evolution of synthetic virology by which harmless recombinant viruses were generated in laboratories (Mann et al., 1983). Pioneering work from Inder Verma’s lab demonstrated that retroviruses could be used as a tool for integrating and expressing the human hypoxanthine phosphoribosyl transferase (HPRT) gene in HPRT negative human and rodent cells (Dusty Miller et al., 1984). This was a significant advancement in the proof-of-concept process that led to “gene supplementation” studies for various diseases and paved the way for human gene therapy trials for diseases such as the Wiskott–Aldrich syndrome (WAS), metachromatic leukodystrophy (MLD), and adrenoleukodystrophy (ALD) (Dusty Miller et al., 1984; Naldini et al., 1996; Aiuti et al., 2013; Biffi et al., 2013; Verma, 2013; Eichler et al., 2016). Leukemia and myelodysplasia arose in a gammaretroviral gene therapy trial for adenosine deaminase-deficient severe combined immune deficiency (ADA-SCID), representing a setback; however, this side effect led to the development of the self-inactivating (SIN) γ vector (Hacein-Bey-Abina et al., 2014). The SIN vector has a 133 bp deletion in the 3′ long-terminal repeat (LTR) of the viral genome, disrupting the promoter/enhancer function of the LTR and allowing only the internal promoter of the gene to drive its expression. SIN lentiviral vectors showed promising efficacy and an outstanding safety profile, with no reports of insertional oncogene activation.

The development of a lentiviral vector-based gene transfer technology contributed significantly to the advancement of HSPC gene therapy for diseases such as ADA-SCID, X-linked SCID (SCID-X1), WAS, chronic granulomatous disease, and β-hemoglobinopathies (Thrasher and Williams, 2017). Advancements in the various steps of autologous hematopoietic stem cell transplantation, such as HSPC mobilization, purification, and *ex vivo* culture, have further enabled the use of autologous HSPCs for gene therapy (Tucci et al., 2022). Few clinical trials, including those assessing treatments for WAS, have exceeded 10 years of follow-up; these studies have shown that LV integration is genome wide and not specific to certain genomic regions (Magnani et al., 2022). The first HSPC gene therapy product to receive FDA approval is Bluebird Bio’s Zynteglo/beti-cel, a lentiviral vector expressing the β-globin (β^A−T87Q^-globin) gene that achieved red blood cell transfusion independence in β-thalassemia patients.

The invention of gene editing technology using programmable nucleases, such as Zinc finger nucleases (ZFNs), transcription activator-like effector nucleases (TALENs), mega nuclease, and clustered regularly interspaced short palindromic repeats/CRISPR-associated protein 9 (CRISPR/Cas9), represents a paradigm shift in therapeutic genome engineering (Urnov et al., 2005; Cong et al., 2013; Mali et al., 2013). These technologies broadened engineering possibilities beyond genetic supplementation to include knockout, targeted insertion, and gene regulation. The nucleases used in the CRISPR genome editing system, guided by target-specific guide RNA, create double-strand breaks or single-strand nicks in the target genomic region, which are repaired by intrinsic DNA repair mechanisms (Chavez et al., 2022). The outcome includes insertion/deletion events (InDels) disrupting the DNA sequence. The DNA repair pathways can also be hijacked by homology-directed repair (HDR) donors, base modifiers (such as base editors), and reverse transcriptases (such as in prime editing) that are fused with Cas9 to incorporate genome modifications (Komor et al., 2016; Anzalone et al., 2019).

Gene editing-mediated HSPC gene therapy has reached clinical studies for blood disorders. In addition to blood disorders, HSPCs are being gene edited for metabolic disorders and neurodegenerative disorders, where a hematopoietic lineage is being used to deliver functional protein at supra-physiological levels (Antony et al., 2022; Buffa et al., 2023). Gene editing requires the delivery of the gene editing cargo, such as nucleic acids and proteins, to the target cells (Wu et al., 2019). However, the efficient and non-toxic delivery of gene editing tools is a major challenge in HSPC gene editing. The adaptability of the delivery systems for large-scale HSPC manipulation is critical for translating HSPC gene editing into clinics. This review summarizes various strategies for delivering gene editing cargos into HSPCs, along with the associated challenges and existing solutions.

### Gene editing cargo for HSPCs: plasmid vs. RNA vs. protein

Plasmids encoding a gene editing nuclease and single guide RNA (sgRNA) are easy to be propagated in large quantities and allow the selection of stably integrated cells using selectable markers (Mátés et al., 2009; Xue et al., 2009). However, few reports on plasmid DNA-mediated gene editing in HSPCs are currently available. An early study revealed that electroporation of ZFN plasmids targeting the CCR5 locus disrupted 17% of the total alleles (Holt et al., 2010). Subsequent studies showed that HSPCs that received plasmid DNA have defective engraftment potential (Mandal et al., 2014). Although the toxicity associated with the electroporation of naked DNA may be due to DNA sensors, such as cyclic GMP-AMP synthase (cGAS)-mediated interferon type 1 (IFN 1) induction, the random integration of plasmid DNA and long-term expression of gene editing components are also of potential concern when using plasmid DNA as a cargo (Lefkopoulos et al., 2020).

One of the core components of CRISPR-based gene editing is guide RNA (gRNA) that navigates the Cas9 nuclease to the target locus. gRNA is mostly delivered as single guide RNA (sgRNA) or as a combination of TracrRNA and CRISPR RNA. HSPCs are sensitive to *in vitro* transcribed (IVT) guide RNAs since cytoplasmic RIG-I detects the same RNA and induces a type 1 IFN response, compromising cell viability (Wienert et al., 2018). The immune sensing of guide RNA can be mitigated by the excision of 5′-triphosphate and the introduction of chemical modifications, such as 2′-O-methyl (M), 2′-O-methyl 3′phosphorothioate (MS) or 2′-O-methyl 3′thioPACE (MSP), at three terminal nucleotides at both the 5′ and 3′ ends (Hendel et al., 2015; Wienert et al., 2018). The gene editing cargo for the CRISPR system, such as Cas9 nuclease in the form of mRNA, is gaining popularity due to recent advances in the mRNA field and the emergence of larger gene editors, such as base editors (size: ∼4.8 kb) and prime editors (size: ∼6.7 kb) that possess effector domains fused to Cas9 variants (Jiang et al., 2020; Liu et al., 2021). Although recombinant protein delivery of base editors has been reported, the production of the recombinant protein is technically challenging (Jang et al., 2021; Knipping et al., 2022). ZFN, TALEN, Cas9, and base editor mRNA result in high editing efficiency (>80%) in HSPCs at various target regions (Genovese et al., 2014; Lattanzi et al., 2019; Newby et al., 2021). In addition, chemically modifying gene editing mRNA improves gene editing efficiency (Vaidyanathan et al., 2018; Jiang et al., 2020). mRNA-mediated gene editing has not been reported to affect the long-term multilineage reconstitution potential of HSPCs (Everette et al., 2023). mRNA delivery is associated with Cas9/gene editor translation by HSPC machinery and the formation of a complex with sgRNA *in situ*. In addition, the mRNA cargo system simplifies multiplex editing since a single gene editor mRNA can be co-delivered with multiple sgRNAs. In contrast to delivering the sgRNA as cloned plasmids, synthesized and chemically modified sgRNA, when used as a ribonucleoprotein (RNP), provides enhanced gene editing due to the improved sgRNA stability. The CRISPR/Cas9 system RNP complex shows highly efficient nuclear delivery, reaching the target within hours of delivery and fading within 48 h (Lattanzi et al., 2019). Additionally, RNPs do not affect the viability, stemness, and engraftment potential of HSPCs (Kim et al., 2014). The rapid clearance of RNPs from the cells results in a relatively high on-target to off-target editing ratio, facilitating selection-free HSPC gene editing (Kim et al., 2014). Near complete editing of therapeutic loci, such as the BCL11a erythroid enhancer and CCR5, has been reported using this technique (Dever et al., 2016; Demirci et al., 2020; Karuppusamy et al., 2022), indicating the efficiency of the approach. The range of gene editing efficiencies using major delivery strategies is listed in Table 1.

**TABLE 1:** Range of gene editing efficiencies reported using major editing/delivery strategies.

S. no.	Conditions	Type of gene editing cargo	Method of delivery	Efficiency of editing	Cell viability	References
1	β-Hemoglobinopathies	Cas9 RNP	Electroporation	68.9%–82.6% in patient cells	NA	Frangoul et al. (2021)
2	Friedreich’s ataxia	Cas9 RNP	Electroporation	39.8%–61.9%	>75%	Rocca et al. (2020)
3	Sickle cell disease	Cas9 RNP and AAV6 donor template	Electroporation	Up to 50% HDR in patient SCD cells	>75%	Dever et al. (2016)
4	Wiskott–Aldrich syndrome	Cas9 RNP and AAV6 donor template	Electroporation	Up to 60% HDR in patient cells	>60%	Rai et al. (2020)
5	Sickle cell disease	Cas9 RNP and ssODN	Electroporation	>20% HDR	>70%	Magis et al. (2022)
6	Sickle cell disease	Adenine base editor mRNA	Electroporation	80%	>90% in HEK 293T	Newby et al. (2021)
7	Beta thalassemia	ABE-RNP	Electroporation	52.9%–77.6%	>80%	Liao et al. (2023)
8	Human immunodeficiency virus type I (HIV I)	CBE-mRNA	Electroporation	Up to 68%	>80%	Knipping et al. (2022)
9	Beta thalassemia	CBE RNP	Electroporation	43%–63.6%	83%	Zeng et al. (2020)
10	Sickle cell disease	Prime editor mRNA	Electroporation	42%	60%	Everette et al. (2023)
11	Human immunodeficiency virus	ZFN mRNA	Electroporation	72.9%	>70%	DiGiusto et al. (2016)
12	β-Hemoglobinopathies	Cas9 RNP	Transmembrane internalization assisted by membrane filtration (TRIAMF)	61.3% ± 4.6%	58.7 ± 11%	Yen et al. (2018)
13	To protect hematopoietic cells from anti-CD33 treatments in acute myeloid leukemia (AML) patients	Cas9 RNP	Polymeric nanoparticle	85%	>86%	El-Kharrag et al. (2022)

### Cargo delivery to HSPCs: options available yet limited

**FIGURE 1:**
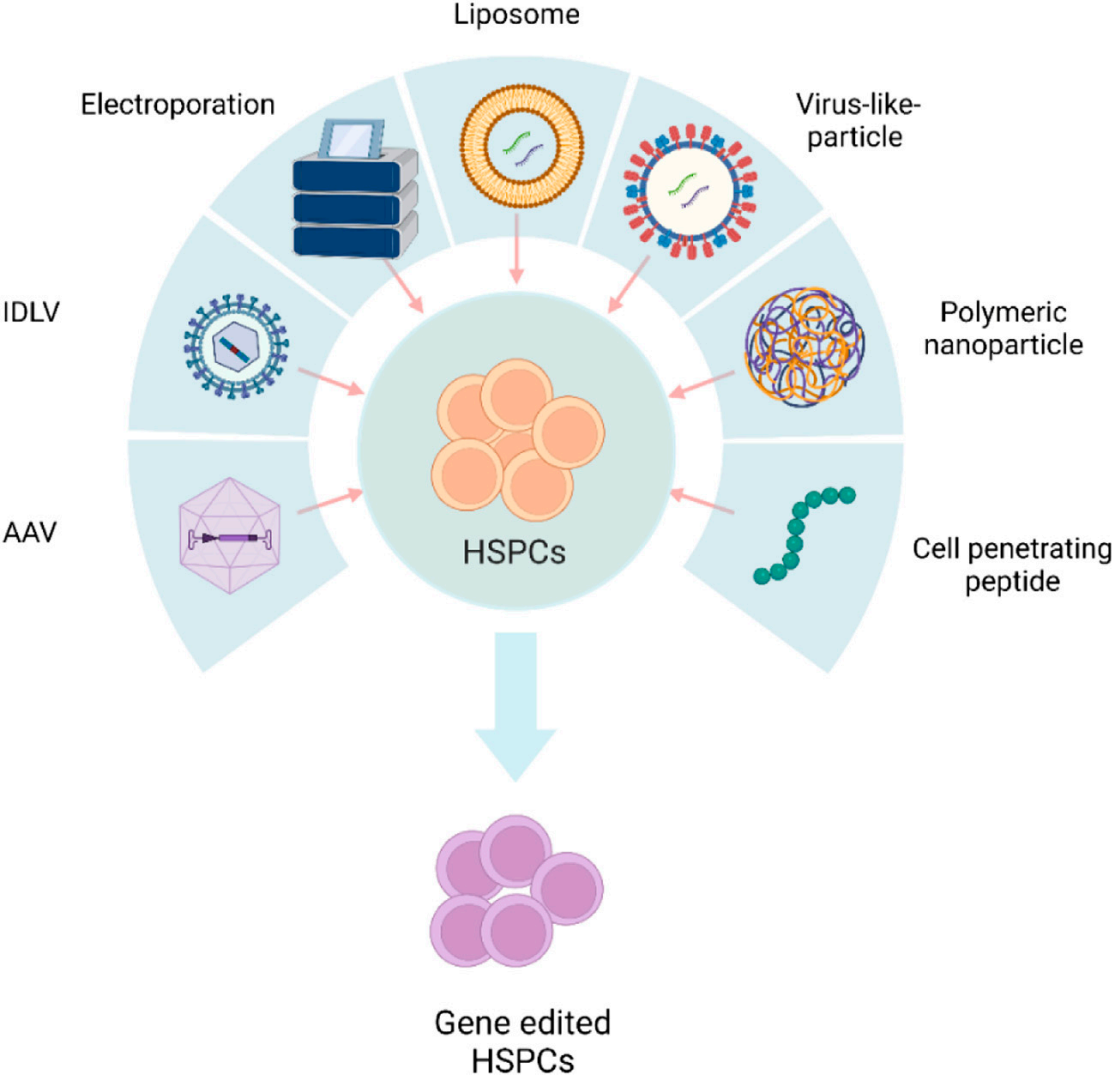
Schematic representation of different delivery systems used in HSPC gene editing. AAV, adeno-associated viral vector; IDLV, integration-defective lentivirus vector. The figure was created using BioRender.

Transient expression of gene editors reduces off-target editing and immune sensing of the gene editors (Kim et al., 2014). The gene editing cargo should preferably be in the form of protein or RNA to achieve “hit and run” genetic modifications. Physical methods, such as electroporation, and chemical methods, including polymeric or lipid nanoparticles and integration-deficient viral vectors (IDLVs), are being explored to deliver biomolecule protein/RNA (Figure 1).

### Electroporation

Electroporation is the transient loss of semi-permeability of the membrane when subjected to an electric pulse, resulting in ion leakage, metabolite escape, and cargo uptake by the cells (Tsong, 1991). Electroporation is the most effective method for delivering mRNA and protein with high efficiency (Vakulskas et al., 2018; Yudovich et al., 2020). In addition, this approach permits multiplex gene editing by delivering RNPs/mRNA to edit different target loci simultaneously (Bak et al., 2017; Zeng et al., 2020; Venkatesan et al., 2023). HDR gene editing requires donor DNA co-delivery with a nuclease and sgRNAs. Recent findings have demonstrated that electroporation could effectively co-deliver all three components, including ssODN, to mediate HDR in HSPCs (Schiroli et al., 2019). A major advantage of electroporation is the ability to deliver larger constructs, such as Cas9 fusion proteins, base editors, and prime editors. Although studies have demonstrated transcriptional changes in HSPCs in response to electroporation, its impact on stemness and functional activity is minimal (Cromer et al., 2018). Numerous electroporation devices, including the Lonza 4D-Nucleofector, the MaxCyte electroporation system, the Neon electroporation system, and the Harvard Apparatus/BTX ECM600 Electro Cell Manipulator/apparatus, are currently being used for HSPC gene editing (Vakulskas et al., 2018; Métais et al., 2019; Frangoul et al., 2021; Magis et al., 2022; Peterson et al., 2022). More importantly, ongoing gene editing-based clinical trials are using electroporation to deliver ZFNs, Cas9, and Cas12a nucleases and ABEs into HSPCs (Table 2). Preliminary reports have demonstrated transfusion independence in previously transfusion-dependent thalassemia and SCD patients (Frangoul et al., 2021).

**TABLE 2:** List of clinical trials using gene-edited hematopoietic stem and progenitor cells for various genetic and acquired diseases. Data were taken from https://www.clinicaltrials.gov.

Clinical trial registry numbers	Conditions	Intervention	Sponsor	Cargo	Mode of delivery
NCT05444894 (I/II)	• Transfusion-dependent beta thalassemia	EDIT-301	Editas Medicine	Cas12a-RNP targeting gamma globin promoter	Electroporation
	• Hemoglobinopathies				
	• Thalassemia major				
	• Thalassemia intermediate				
NCT05456880 (I/II)	Sickle cell disease	BEAM-101	Beam Therapeutics Inc.	Adenine base editor (ABE)	Electroporation
NCT04774536 (I/II)	Sickle cell disease	CRISPR_SCD001	Innovative Genomics Institute (IGI)	Cas9 RNP with ssODN-targeting HBB gene	Electroporation
1. NCT03655678 (II/III)	Sickle cell disease and beta thalassemia	CTX001	Vertex Pharmaceuticals and CRISPR Therapeutics	Cas9 RNP-targeting BCL11A	Electroporation
2. NCT04819841 (I/II)	Sickle cell disease	GPH101	Graphite Bio	Hi-Fi Cas9 RNP with AAV6-targeting HBB gene	Electroporation
3. NCT04925206 (I)	Transfusion-dependent beta thalassemia	ET-01	EdiGene (Guangzhou) Inc.	Cas9 mRNA and sgRNA	Electroporation
4. NCT02500849	Human immunodeficiency virus infection	SB-728mR	City of Hope Medical Center	ZFN mRNA-targeting human CCR5 gene	Electroporation using the MaxCyte GT transfection system
5. NCT03745287 (II/III)	Sickle cell disease	CTX001	Vertex Pharmaceuticals and CRISPR therapeutics	Cas9 RNP-targeting BCL11A	Electroporation

**FIGURE 2:**
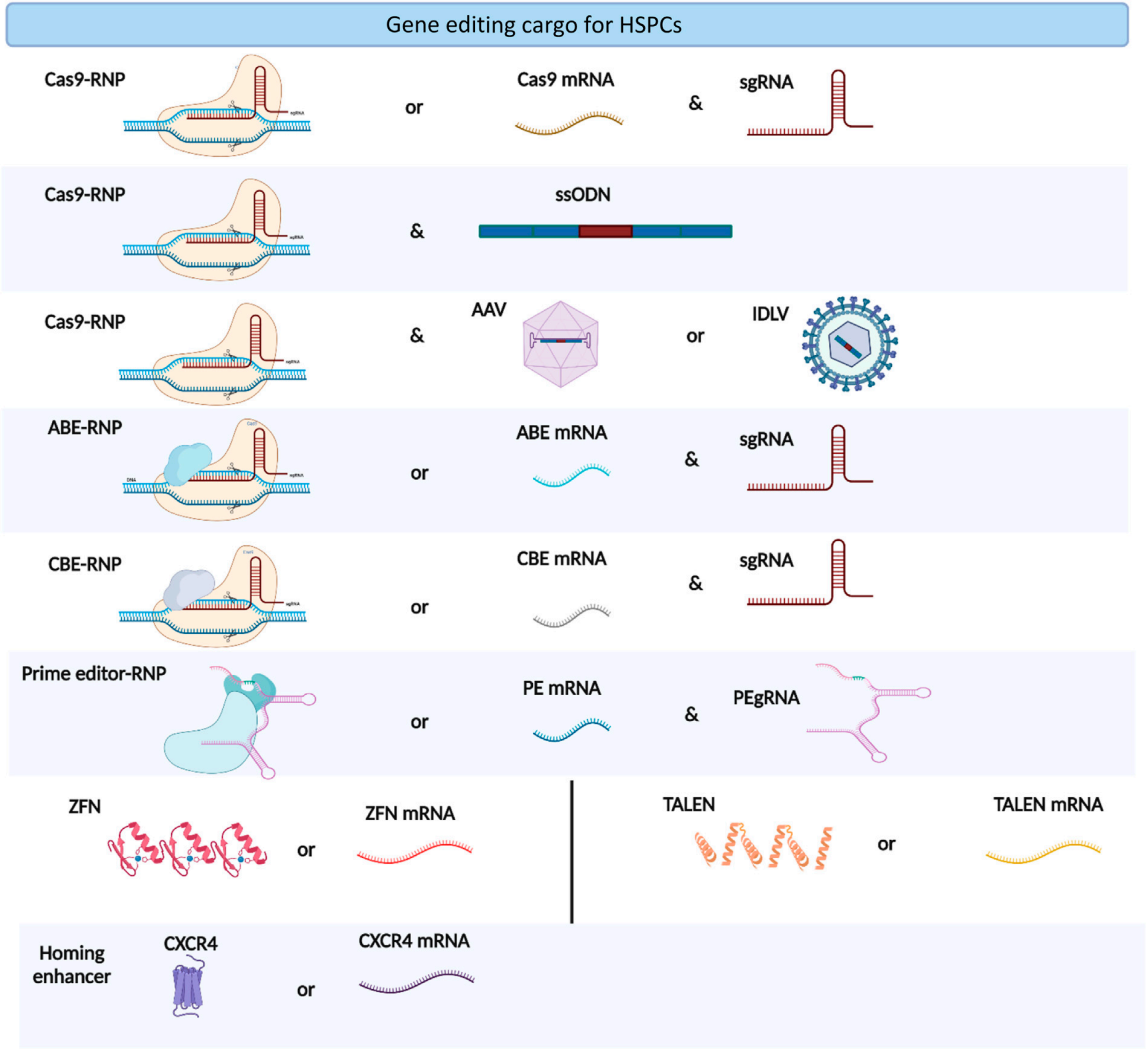
Types of gene editing cargoes used in HSPC gene editing. The figure was created using BioRender.

The co-electroporation of beneficial factors, such as homing enhancers and p53 suppressors, with gene editing cargos is a new approach to enhance the potential of gene manipulation (Figure 2). Specifically, the electroporation of CXCR4 mRNA increased the homing capabilities of HSPCs in xenograft models (Omer-Javed et al., 2022). Similarly, the co-electroporation of GSE56, a dominant negative p53, overcomes the proliferation delay induced by gene editing reagents and improves the frequency of long-term repopulating edited HSPCs (Schiroli et al., 2019). Electroporation also enhances viral vector delivery into HSPCs by enhancing cellular endocytosis (Charlesworth et al., 2018). The electroporation-assisted enhanced transduction improves donor delivery, thereby increasing the HDR gene editing frequency.

### Mechanical delivery of gene editing cargos

Quiescent HSCs exhibit reduced endocytosis; thus, endocytosis-independent alternative delivery methods should be explored (Kolb-Mäurer et al., 2002). Cell constriction-driven membrane deformation induces the entry of biologics into mammalian cells. To this end, Jonathen Yen *et al.* developed a “transmembrane internalization” method assisted by membrane filtration (TRIAMF) to deliver CRISPR RNPs into human HSCs, observing up to 60% editing at γ-globin loci (Yen et al., 2018). In this method, the RNP and HSPC mixture was loaded into a 3 mL reservoir syringe and pushed through the membrane using 5 pounds per square inch (PSI) nitrogen pressure. TRIMAF-edited cells retained *in vivo* engraftment potential, and the cell recovery was significantly better than that achieved with standard electroporation-mediated editing (Yen et al., 2018). The advantage of this method is that HSPCs can be edited in the culture medium without the use of electroporation buffers, which will considerably reduce cellular stress. The improved recovery of TRIMAF-edited cells suggests that filtration-related stress on HSPCs is less severe than electroporation-associated stress.

Acoustofluidic sonoporation is another mechanical delivery method. This approach uses acoustic waves to induce transient pores and membrane permeabilization; the intracellular delivery of GFP plasmid DNA into HSPCs was achieved with an efficiency of 20% and resulting cell viability of 92% (Belling et al., 2020). Volume exchange for convective transfection (VECT) was used to deliver GFP mRNA into HSPCs and demonstrated 70% delivery efficiency with 80% cell viability (Loo et al., 2021). An alternative mechanical transfection for the delivery of RNA is hollow aluminum oxide tubes known as nanostraws, which are packed with biomolecules (DNA, RNA, and dextran). This approach uses a direct fluidic pathway from a cargo-containing compartment beneath the nanostraw membrane to deliver the cargos into the cytoplasm. Nanostraws delivered mRNA-encoding GFP into the cytoplasm of CD34^+^ cells at an efficiency of around 75% and did not elicit any inflammatory responses in HSPCs, unlike electroporation (Schmiderer et al., 2020). Although the mentioned systems are potential alternatives to electroporation for the delivery of gene editing cargos into HSPCs, their translational prospects have yet to be explored.

### Nanoparticles

Cationic/ionizable polymeric nanoparticles (PNPs) and lipid nanoparticles (LNPs) can encapsulate negatively charged nucleic acids and deliver the content into the cells through various endocytosis mechanisms. These nanoparticles are easy to construct, less immunogenic, non-invasive, suitable for transient gene expression, and customizable for cell/tissue-specific delivery; thus, nanoparticles are ideal for delivering various designer nucleases (Cruz et al., 2022).

CD44 siRNA-encapsulated PNPs successfully silenced the expression of CD44 protein in CD34^+^ AML cells, demonstrating that HSPCs can be manipulated using PNPs (Gul-Uludağ et al., 2014). Similarly, α-CD105-decorated PNPs efficiently delivered GFP mRNA at an efficiency of ∼50% and demonstrated improvement in the CD34+CD133+ HSPC count when delivering musashi-2 mRNA (Moffett et al., 2017). PNPs comprising optimized poly β-amino esters (PBAE-PNP) were used to pack Cas9 RNPs and mediate gene editing in HSPCs at an efficiency of 70% when targeting CD33 and the HBG promoter (El-Kharrag et al., 2022). PNPs interact with HSPCs within 2 hours of co-culture and do not compromise cell viability. This rapid interaction has the potential to reduce the *ex vivo* culture duration, which in turn reduces the culture-associated stress to HSPCs when performing an *ex vivo* editing approach. Furthermore, PNP-mediated gene-edited HSPCs demonstrated long-term engraftment with multilineage potential comparable to that observed after traditional electroporation. In comparison with electroporation, a three-fold lower Cas9 RNP dose was sufficient to achieve similar gene editing efficiency, indicating that PNPs could be a preferable alternative to electroporation (El-Kharrag et al., 2022). Although PNPs offer the option of repeated addition to culture media to improve editing efficiency and multiplex gene editing, these options have not yet been evaluated.

### Virus-like particles

Although LV-mediated delivery of gene editing cargos in HSPCs is efficient, the persisting expression of Cas9-gRNA can lead to adverse effects, which is a major concern in clinical application. The delivery of gene editing nucleases as RNPs is a better alternative; however, this approach demands an efficient system (electroporation or PNPs) for intracellular delivery (Zhang et al., 2020). In scenarios where transient expression is required, engineered virus-like particles (eVLPs) can replace viral vectors, overcoming the above mentioned limitations. VLPs can be produced from several viruses and engineered to encapsulate gene editing nucleases by fusing with viral proteins (for example, Cas9 fused with VSV-G or Gag). During the self-assembly of viral proteins, gene editing nucleases can be encapsulated into the VLPs as an RNA or protein. HIV-1 Gag-Cas9 VLPs displayed ∼14% editing efficiency at the CCR5 locus with reduced off-target activity in TZM-bl cells (Choi et al., 2016). Baboon envelope pseudotyped VLPs (known as nanoblades) resulted in ∼50% gene editing efficiency in cord blood-derived CD34^+^ HSPCs (Mangeot et al., 2019). Base editors have also been packed inside VLPs and delivered *in vivo* into the mouse liver*,* resulting in an editing efficiency of ∼63% (Banskota et al., 2022). VLPs can be custom-made to specifically edit CD4 T cells by the surface display of the R5-tropic HIV envelope (Hamilton et al., 2021; Hamilton et al., 2022). This approach provides an opportunity to make cell or tissue-specific VLPs for *in vivo* gene editing. However, the efficiency of VLP-mediated gene editing in HSPCs has yet to be elucidated.

### Cell-penetrating peptides

The discovery of the cell penetration property of the trans-activator of transcription (TAT) protein in HIV has shed light on the peptide sequences that can traverse the plasma membrane (Frankel and Pabo, 1988). TAT has an 11-amino acid cationic peptide sequence that mediates intracellular delivery (Green and Loewenstein, 1988) and has opened new avenues for cargo delivery. The lysine and arginine residues in the peptide sequence mediate the intracellular delivery of biomolecules by interacting with the plasma membrane (Ter-Avetisyan et al., 2009). Prior findings also demonstrated that cell-penetrating peptides (CPPs) could modulate endocytic mechanisms for higher internalization of the biomolecules (Pae et al., 2014). CPPs are typically 5–30 amino acids long and cationic, allowing them to covalently and non-covalently bind to nucleic acids. To date, over 1800 CPPs have been reported (https://webs.iiitd.edu.in/raghava/cppsite/) and are being explored broadly in *in vitro* and *in vivo* applications due to their good safety profiles and low immunogenicity.

In addition to protein-derived CPPs, synthetic peptides with chimeric sequences from two different proteins can be used to facilitate intracellular delivery (Gao et al., 2010). Screening peptides using a phage random peptide library predicted unique peptides for specific delivery into colorectal cancer cells, showing that CPPs can also be explored for targeted delivery (Wang et al., 2012). The major advantage of CPPs is the simplicity of forming a complex with the cargo that can be supplemented into the culture medium.

CPP-mediated delivery of functional molecules into HSPCs has also been reported. Specifically, TAT-fused NF-Ya, a subunit of the NF-Y transcription factor, penetrated HSPCs successfully and activated its target, HOXB4, promoting a 4-fold increase in HSPC engraftment (Domashenko et al., 2010; Wang et al., 2012). Multilineage reconstitution was also improved by the direct fusion of HOXB4 and TAT (Lee et al., 2013). Similarly, TAT-fused polycomb protein reportedly improved long-term stem cell reconstitution (Codispoti et al., 2017). In another interesting study, a synthetic peptide, PepFect14, was used to tag the Cas9 RNP complex and could perform gene editing at an efficiency of >70% in HEK 293T cells (Wang et al., 2012). The efficiency of PepFect14-tagged Cas9 RNP in mediating gene editing in HSPCs has not been evaluated; however, two very recent works showed that the fusion of TAT with the HA2 endosomolytic peptide enabled the delivery of Cas9 RNPs into T cells and HSPCs at a high frequency. Both studies showed minimal perturbation of the transcriptome profile after chimeric peptide delivery, indicating the enhanced viability of primary cells when using this approach (Foss et al., 2023; Zhang et al., 2023).

### Adeno-associated viral vectors

Adeno-associated viral vectors (AAVs) are the most successful vectors for delivering genes in systemic settings. Multiple AAV-based gene therapy products are approved by the FDA for clinical use. The potential to infect both dividing and non-dividing cells, stable transgene expression, and safety profile of AAVs makes them a prominent vector for *in vivo* gene therapy. AAVs have been used for a range of pathological conditions, including inherited retinal dystrophy and hemophilia (Li and Samulski, 2020). Roctavian, an AAV-carrying clotting factor VIII, is a recently approved gene therapy product for hemophilia A.

There are at least 10 AAV serotypes, each with tropism to specific cell types. Among these serotypes, AAV6 has a high tropism for HSPCs, and the transduction efficiency can be improved by using capsid-modified AAV6 vectors (Büning and Srivastava, 2019). In HSPC gene therapy, AAV6 is often used for HDR donor delivery. Electroporation of Cas9 RNP followed by AAV6 transduction and enrichment resulted in 90% targeted integration (Dever et al., 2016). AAV6-mediated donor delivery into HSPCs was successfully tested for the correction of many diseases, including SCD, WAS, SCID, and lysosomal storage disorders (Pavel-Dinu et al., 2019; Rai et al., 2020; Scharenberg et al., 2020; Wilkinson et al., 2021). AAV6 donors can be combined with ZFNs, TALENs, and Cas9. Cas9-AAV6-mediated HBB correction resulted in long-term stable hemoglobin A (HbA) expression and decreased sickle hemoglobin (HbS) expression in the Townes-SCD mouse model (Wilkinson et al., 2021). AAV6 used as a donor also demonstrated a highly efficient monoallelic insertion frequency of up to 94% at the HBB locus across hematopoietic cell lines (Dever et al., 2016). A triple mutation in the AAV6 capsid and the transduction at high cell confluency can transduce more than 90% of HSPCs (Ling et al., 2016) AAVs have advantages over LVs because of their non-integrating viral DNA; however, applications are limited due to the low cargo-loading capacity of 4.7 kb. Recently, this limitation has been eliminated by separating the large transgene into two AAV vectors or by using an oversized AAV vector (Bak and Porteus, 2017; Zhi et al., 2022). Immune responses against AAV-transduced cells are another limiting factor for AAV-mediated gene therapy (Mingozzi and High, 2011).

### Integration defective lentiviral vectors

The current third-generation LV vectors are self-inactivating and, on transduction, stably integrate into both dividing and non-dividing cells. These vectors have a large packaging capacity of up to 9 kb (Kumar et al., 2001). Viral integration into the HSPC genome is not associated with significant activation of DNA repair machinery; however, nuclear accumulation of vector DNA induces the ATM-P53-p21 signaling cascade, delaying the proliferation of primitive HSCs (Piras et al., 2017). The transient activation of the DNA damage response does not affect HSPC engraftment or lineage distribution. Although it is difficult to include all the native regulatory elements in the transgene construct, LV vectors drive effective gene expression using native or artificial promoters and a few enhancer sequences. The short-term presence of the gene editing nuclease is important to reduce the chances of deleterious effects caused by off-target editing; hence, researchers introduced an integration defective lentiviral vector (IDLV) using a trans-complementing packaging construct with a point mutation at the integrase moiety. The integrase protein of IDLV recruits the host cellular protein LEDGF/p75 to mediate the HDR in hESCs (Wang et al., 2017). IDLV vectors have also demonstrated successful HDR in HSPCs, where they provoke a minimal DNA damage response and demonstrate a lower entrapment of the viral sequence at the break site than AAV6 (Ferrari et al., 2022).

### Enhancers of cargo delivery

Despite the demonstration of successful delivery of cargo into HSPCs, the delivered concentrations may be insufficient to achieve therapeutic benefits due to interpatient heterogeneity, transgene size, or the requirement for a vast number of gene-modified cells for disease reversal. It is possible to scale up the quantity of cargo delivered utilizing high multiplicities of infection or two-hit transduction. However, manufacturing large doses of viral vectors is expensive and laborious. Several groups have been working to identify the conditions that maximize cargo delivery.

Different small-molecule enhancers for LV transduction in HSPCs have been reported, including rapamycin, prostaglandin E2 (PGE2), LentiBOOST, and cyclosporin H (CsH) (Wang et al., 2014; Heffner et al., 2018; Petrillo et al., 2018; Schott et al., 2019). Compound screening with the SCREEN-WELL^®^ FDA-approved drug library identified prostaglandin E2 as a positive regulator of vector copy number (VCN). In a comparative study, LentiBOOST treatment increased the VCN 2-fold (Petrillo et al., 2015). CsH was found to boost transduction levels by blocking IFITM3, an innate immune factor that restricts LV entry (Wang et al., 2014; Heffner et al., 2018; Petrillo et al., 2018; Schott et al., 2019). In line with this, caraphenol A, a resveratrol trimer, has been demonstrated to inhibit both IFITM2 and IFITM3, enhancing transduction efficiency (Ozog et al., 2019).

In addition to their ability to enhance transduction efficiency, the safety profile of enhancers must be considered, as some molecules negatively affect HSPCs. Cyclosporin A (CsA) and rapamycin cause a delay in proliferation, while PGE2 reduces the CD34^+^ CD90^+^ primitive cell population (Wang et al., 2014; Petrillo et al., 2018; Schott et al., 2019). We have shown that culturing HSPCs with a cocktail of small molecules, resveratrol, UM171, and SR1 increases the frequency of gene-modified stem cells (Christopher et al., 2022; Karuppusamy et al., 2022). Vesicular stomatitis virus envelope glycoprotein (VSV-G) pseudotypes from wild-type HIV provide a powerful tool for cargo delivery (Duvergé and Negroni, 2020). Low-density lipoprotein (LDL) is the receptor for VSV-G, and its low levels in quiescent HSCs limit the VSV-G pseudotyping approach (Lévy et al., 2015). LVs pseudotyped with the Baboon or measles virus envelope have also shown improved cargo delivery into long-term HSCs (Girard-Gagnepain et al., 2014; Lévy et al., 2017).

Alt-R, an electroporation enhancer, is a short, single-stranded deoxyoligonucleotide (ssODN) that has no homology with the human genome and was used to increase the efficiency of Cas9 RNP electroporation and gene editing in human CD34^+^ HSPCs (Shapiro et al., 2020). Increased gene editing efficiency was also reported when a high dose of sgRNA was used in RNP complex formation or when RNPs were electroporated in the presence of anionic polymers, such as poly-l-glutamic acid (Nguyen et al., 2020). All these strategies aim to improve stability by preventing Cas9 aggregation. In addition to the enhancers of cargo delivery, there are small molecule compounds that can improve the outcomes of gene editing (Azhagiri et al., 2021; Selvaraj et al., 2023).

### *In vivo* targeting of HSPCs

The current HSPC gene therapy protocol involves myeloablative conditioning with alkylating drugs for the efficient engraftment of the genetically manipulated cells. The conditioning regimen related toxicity limits the application of HSPC gene therapy (Sean Burns, 2021). In addition, the *ex vivo* culture and manipulation of HSPCs are laborious processes that require expensive reagents, a GMP facility, an on-site stem cell transplantation facility, and trained personnel. Recently, *in vivo* targeting of HSPCs has gained attention as it overcomes the complications associated with *ex vivo* manipulation. Although gene-modified HSPCs are considered the drug product in *ex vivo* HSPC gene therapy, cargo/gene editor-packed delivery vectors are the drug product in *in vivo* gene therapy. Cargo-packed delivery reagents are easier to characterize for regulatory approval, scale-up, off-site preparation, and transport.

HSPCs are highly heterogenous; thus, targeting HSPCs *in vivo* is a challenging gene editing approach. Anti-CD117 and CD45 antibodies can specifically target HSPCs and mediate receptor internalization (Castiello et al., 2021; Russkamp et al., 2021). AMG 191, a clinical-grade CD117 antibody, depleted myelodysplastic syndrome (MDS) HSCs in mouse models and is currently being evaluated in clinical trials for pediatric SCID (De Franceschi et al., 2019). Saporin- or amanitin-conjugated CD117 antibodies have been shown to internalize into HSPCs and inhibit ribosome or RNA polymerase II, respectively, thereby depleting the HSPCs and creating a space for donor cell engraftment (De Franceschi et al., 2019; Pearse et al., 2019). Saporin-conjugated CD117-mediated niche clearance has been demonstrated in mouse studies, non-human primate (NHP) studies, and immunodeficient mouse models for human HSPC engraftment. Intriguingly, a high level of HSPC engraftment post-CD117 conditioning was observed in a murine hemophilia A gene therapy model (Gao et al., 2019). This approach may provide a promising alternative to overcome neutropenia, anemia, and lymphocytopenia associated with the existing cytotoxic conditioning regimen that causes collateral damage to the immune system (Hartman et al., 1998).

Adenoviral vectors (AdVs) have been previously explored for HSPC targeting and gene/base editing *in vivo*. Ad5/35++ and Ad5/F3+ have a tropism toward the CD46 and DSG2 receptors present in HSPCs (Wang et al., 2022). AdV packed with gene editing cargos (Cas9 or base editors) was intravenously administered after mobilizing HSPCs into the peripheral blood (Li et al., 2022a). This approach resulted in a manipulation efficiency of up to 6% in LT-HSCs (CD34^+^CD90^+^CD45RA-) *in vivo* in NHP models. The selection of gene-modified cells resulted in a high frequency of γ-globin-expressing red blood cells (Li et al., 2022b). A similar approach in the CD46/β-YAC mouse model showed 60% base conversion (Li et al., 2021). However, the gene-modified HSPCs had poor bone marrow homing efficiency, which could be improved by the co-delivery of CXCR4 (Felker et al., 2022).

Another intriguing approach is LNP-mediated targeting of HSPCs for *in vivo* delivery. This approach is challenging since HSPCs are surrounded by bone marrow stromal cells. Krohn et al. reported that siRNA-encapsulated nanoparticles comprising C15 epoxide-terminated lipids and low-molecular-weight polyamines could deliver cargos into the HSC niche and modify the hematopoietic process (Krohn-grimberghe et al., 2021). Similarly, preliminary data from Intellia Therapeutics showed *in vivo* gene editing of HSPCs up to 40% in a dose-dependent manner (Sean Burns (Intellia), 2021). Another interesting study demonstrated that a CD90 antibody-decorated LNP (lipid nanoparticle) targets the bone marrow CD34^+^ cells with an efficiency of 4% (Cannon et al., 2021). In addition, LNPs decorated with CD117 antibodies could deliver mRNA encoding Cre-recombinase into HSPCs *in vivo* (Breda et al., 2023; Shi et al., 2023). Thus, decorating LNPs (encapsulated with nucleases encoding mRNA) with HSPC-specific antibodies is a viable strategy for modifying the targeted HSC population. Intraosseous injection of lentiviral vectors efficiently transduced HSPCs *in vivo* up to 10%. Interestingly, the transplantation of *in vivo* transduced HSPCs into a secondary recipient showed transgene expression as measured by the correction of the bleeding phenotype in hemophilia A (Joo et al., 2022). Another study showed that intraosseous injection of supramolecular nanoparticles (SMNPs) *in vivo* resulted in gene knock-in of up to 2% (Ban et al., 2022). Overall, *in vivo* HSPC-targeted gene editing is a promising approach for HSPC gene therapy. However, considerable advances are needed to further improve efficiency for therapeutic application. The native and adaptive immune responses against delivery vectors, Cas9, and sgRNA, as well as the repercussions of off-target tissue editing, are among the biggest hurdles to be addressed (Singh et al., 2018; Charlesworth et al., 2019; Wang et al., 2023).

### Challenges with the existing delivery platform and future directions

Current HSPC gene therapy uses an *ex vivo* manipulation approach that involves immunomagnetic separation of HSPCs, pre-stimulation with a cytokine-enriched medium, and electroporation of the gene editing reagents. These procedures must be conducted in a facility that adheres to current good manufacturing practices (cGMP) to reduce the hazards of microbiological contamination. The procedures make the therapy prohibitively expensive, particularly burdening patients from low- and middle-income countries. Although *in vivo* gene therapy for HSPCs offers promising and potential advantages to overcome the limitation of *ex vivo* therapy, the lack of efficient delivery tools that can specifically target HSPCs *in vivo* and mediate gene editing at high efficiency remains to be resolved.

The efficiency of cargo delivery, the impact of delivery on the biology and function of HSPCs, the feasibility of the delivery platform for manipulation on a clinical scale, and the cost associated with the delivery platforms are critical limitations for translating pre-clinical HSPC gene therapy studies. A closed and automated HSPC gene editing platform that performs purification, culture, and editing with minimum user interface would eliminate the requirement for a GMP facility and specialist human resources, lower costs, and make HSPC gene therapy more affordable for a broader patient population.

Electroporation is the current gold standard for gene transfer into HSPCs. However, electroporation requires copious quantities of gene editing cargo. Recent findings demonstrated that LNPs could mediate the same level of gene editing with 3-fold fewer reagents (El-Kharrag et al., 2022). LNP-mediated editing decreases the cost and reduces off-target gene editing and mechanical stress on HSPCs. In line with this, it was found that LNP-edited HSPCs engraft better than electroporated HSPCs in NSG mice, which was attributed to greater cell recovery. Importantly, RNP-encapsulated LNPs can be manufactured and transported in a lyophilized form. Although there are few LNPs available to facilitate HSPC gene editing (El-Kharrag et al., 2022), significant efforts could further improve the efficiency and specificity of this approach. *In vivo* gene editing with LNPs, antibody-coated LNPs, or VLPs may further improve the efficiency and specificity of HSPC gene therapy. This strategy may permit repeated dosing for enhanced efficacy. However, multiple other concerns, including immunological responses to LNPs and VLPs, accumulation of LNPs/VLPs in the liver and spleen, and the stability of the cargo in the bloodstream, need to be addressed. A recent study demonstrated that a new protein delivery system derived from the extracellular contractile injection system of endosymbiotic bacteria can deliver protein payloads, such as Cas9 and base editors, to human cells (Kreitz et al., 2023). The efficiency of this system in delivering gene editing reagents to HSPCs and its potential use for targeting HSPCs *in vivo* remains to be investigated.

## Conclusion

HSPC gene editing therapy aims to permanently correct the disease, and the approach has shown great promise in a phase I/II clinical study for β-hemoglobinopathies and in pre-clinical studies for several other diseases. This success has encouraged the testing of HSPC gene editing for infectious diseases, such as HIV, and non-hematological diseases, such as metachromatic leukodystrophy and mucopolysaccharidosis type I. The development of mobilization-based chemotherapy-free HSPC transplantation and concomitant overexpression of homing/engraftment enhancers for early engraftment is expected to improve the success rates of HSPC gene editing (Felker et al., 2022; Omer-Javed et al., 2022). The *ex vivo* culture-free HSPC gene editing and gene editing of a sub-population of HSPCs can potentially simplify the gene therapy approach (Radtke et al., 2020; Karuppusamy et al., 2022; Venkatesan et al., 2023). Gene editing delivery tools have played a substantial role in the success of HSPC gene editing; however, they are also a cause of the high cost of HSPC gene therapy. Recent advances in editing liver cells *in vivo* with LNP showed promising therapeutic outcomes for hereditary transthyretin amyloidosis (hATTR) and other diseases in clinical trials. Considering these advances, we anticipate that LNP-packed gene editing reagents may simplify the process and reduce the cost of HSPC gene therapy. GMP-free HSPC manipulation is a way to reduce the cost and increase access to HSPC gene therapy. The progress in various fronts offers great promise for a cost-effective, phenomenally successful clinical application of HSPC gene editing.
